# Atlanta Academy of Medicine

**Published:** 1877-07

**Authors:** J. S. Todd

**Affiliations:** Reporter


### Reports of Societies.

### ATLANTA ACADEMY OF MEDICINE.

J. S. TODD, M.D., REPORTER.

ATLANTA, June 5, 1877.

The President, Dr. V. H. Taliaferro, in the chair.

The following letter was read and discussed:

(Near) TALLAHASSEE, FLA., May 20, 1877.

*Dr. V. H. Talliaferro, Pres. Atlanta Academy of Medicine:*

DEAR SIR: Knowing the lively interest you take in every thing pertaining to medicine, I would be glad to ask an expression of the Academy, through you, upon the subject of "Morphia as a Parturient." Our text books, rather than attribute such action to it, lay it down as one article the medical properties of which are infallibly to the contrary. This I have learned, by often chagrining experience, is not the case. More than once I have administered it with a view to stopping the parturient process, when, to my astonishment and amazement, it but increased, both in frequency and vehemence, the throes of labor, and hastened the case to a speedy termination.

I will give the history of one case, which is typical of them all. It is as follows: M. C., col., æt. about 27, has had several children. Was taken with labor pains on Friday, accompanied by almost unbearable continuous pain in lumbar region and hips. The old midwife in attendance becoming alarmed, sent for me on the following Sunday at 8 o'clock. Upon reaching the bedside, I found my patient suffering greatly from the pain in parts mentioned above, but there were no true labor pains; they had almost ceased, and the patient was much exhausted. An examination aroused suspicion of impacted head. Chloroform was administered by inhalation, and ergot internally. The pains returned, and after some hours, finding that my suspicions were justly aroused, I performed craniotomy on a dead child with a keen knife, as I had no instruments with me. The pains then ceased, and for hours repeated doses of the usual quantity of ergot were given without the slightest effect. The same use was then made of a decoction of gossypiirvadix, with like effect. The day was now well nigh spent, and as I could devise no means by which to make traction upon the fœtus or cause a return of the pains, I administered a one-third grain dose of morphia, stating at the time that I would go for my instruments, thinking that the patient would go to sleep and that all would be well until my return. I left and returned in one hour, to find that "all was over." The patient and friends stated that about twenty or thirty minutes after I left the house the pain in hips and other parts subsided, and immediately there was a return of severe, genuine labor pains, which expelled the contents of the womb in a very short time. Were this the only case, I would attribute it to a mere return of pains, but this I cannot do, knowing that in every case in which I have given the drug, *after there was considerable dilatation of the os uteri*, it met with a like result.

In searching for a predecessor in this matter, I find that for twenty years or more medical attention has been occasionally drawn to the subject, and some have used morphia in this connection with much success. None, however, have attempted to give its physiological action upon the uterus; nor do I; would merely suggest, that as chloroform allays pseudo pains without interfering with the normal contractions of the womb, may not morphia allay the one and stimulate the other? I do not claim for morphia any such action, except when the os is already much dilated.

Please submit this to the Academy for discusson, as I am anxious to know the opinions and experiences of the learned gentlemen who constitute that body, upon this subject.

Dr. Jno. M. Johnson: Opium arrests the pains of labor for awhile, and this rest to a uterus that has been constantly contracting for any length of time, causes it to gather such additional strength, by reason of the repose afforded it by this anodyne, that when labor does set in again the pains are longer, hence, more effectual. This period of respite also gives time to the os and soft parts to relax, if it does not directly cause relaxation or dilatation of the mouth; for when the longitudinal and circular fibres are not acting conjointly, he believes that opium is our remedy to produce that result, by obtunding the sensibility or contractility of the circular class, without interfering with the longitudinal set, and an opening of the os is the result. He is so firmly of the opinion that opium or its alkaloids are *not* parturient, that it has been his custom, where he saw fit to delay a labor that was progressing with too much rapidity, or there were other reasons to delay its progress, such for instance as the calling of a consulting physician, to give it, and the results obtained confirm his belief. There are circumstances, other than idiosyncrasy, under which opium acts as a purgative.

Dr. J. P. Logan: As a rule, opium arrests regular contractions, and causes the interval between them to be much lengthened; irregular pains are corrected and made regular; it delays the labor. As an evidence of its *not* being parturient, it is confessedly our very best remedy to prevent and arrest an abortion. Where the os is undilated, it will always delay, frequently arrest for the time altogether.

Dr. W. F. Westmoreland: Under certain circumstances, he is of the opinion that, irrespective of the condition of the os, opium will act as indicated by Dr. Logan.

Dr. J. G. Westmoreland agreed with Dr. Logan, and said that Dr. Johnson was correct in saying that indirectly, where the circular fibres of the os refused, by reason of irritability, to relax, that in that case opium was parturient, indirectly. After normal expulsive pains are established, he is not sure that opium will arrest them.

Dr. Grant had been so much impressed by several articles that he had read, claiming parturient properties for opium, that he had feared to give it where he wished a labor not to progress with rapidity. He detailed several cases where recently he had administered it, and the results were negative.

The President said, in tedious cases, where the pains are more pseudo than otherwise, wearing the woman out without accelerating the advance of the child; where sleep has been lost for any length of time, and these conditions setting up a state of nervousness that is distressing, he gives opium to secure rest, and generally gets it; sometimes for as long as five or six hours. If the dose is sufficiently large, the interval between even normal pains will be lengthened. Tetanic, without complete relaxation, call for the use of opium, and by it are converted into regular physiological and rythmical contractions. It undoubtedly corrects certain irregularities and conditions already mentioned by the gentlemen who have preceded him, and this being true, erroneous deductions have from time to time been drawn by various practitioners. He would consider it unfortunate for this dogma to obtain general sanction, for that would deprive the accoucheur of a valuable weapon, and probably lead to the abandonment of a remedy which, under certain circumstances, is invaluable.

Dr. W. F. Westmoreland operated to-day on a gentleman thirty-five years of age, for stone. He called the attention of members to several peculiarities. The stone exhibited was quite small—about the size of a butter bean—although symptoms of its existence had been present for eight years—he about that time having suffered from nephretic colic, the calculus not being voided from the bladder. The opening through perineum had closed so completely that the urine came off by the urethra. The size of the stone, its age, and the water not dribbling, were all unusual.

Dr. Calhoun asked if he did not suspect the existence of another stone in the bladder.

Dr. Westmoreland had explored the bladder with his finger, which on account of the thickness of its walls, causing less capacity, facilitated the thoroughness of the operation of search.

Dr. J. M. Johnson: When there is but one stone, was of opinion that it was more oval and rougher than this one, which was smooth and irregular.

Dr. Westmoreland: That depends on the composition. When they are soft or phosphatic, attrition did cause them to assume the appearance mentioned by Dr. J., but when hard or oxalic, as he thought this one was, they were often, even when not multiple, smooth and undefined in shape.

Dr. Calhoun mentioned a case of encephaloid and melanotic tumor of the orbit, which illustrated the tenacity of life occasionally met with. The first operation for its removal was done a year ago, and he thought he thoroughly removed all the diseased structure; but in six or eight weeks it reappeared, this time filling the cavity of the eye and involving the lower lid especially, and the upper slightly. The operation of removal the second time was performed with great care, and Vienna paste used afterwards. This operation was done early in December last. It reappeared in two weeks. On yesterday he learned that the man, although he had been upon the verge of the grave for three months, was still alive; the cancer now occupying the regions around the eye, the lower half of the face on that side, had extended across the bridge of the nose and to both sides of the lip; there were also evidences of its extension along the optic nerve to the brain. This extensive cancer has existed for six months. The case was originally one of simple pterygium. He believes the exciting cause of the cancer in this case was the repeated applications of nitrate of silver and nitric acid, which were applied to the pterygium to effect a cure, by an attendant.

Dr. J. G. Westmoreland: Does Dr. Calhoun mean to convey the idea that the pterygium was changed to cancer by the application of caustics?

Dr. Calhoun: Where there is a cancerous diathesis, undoubtedly; he was sure that constant unwarrantable interference, that caused continuous local irritation, would lead to its development; and while it was not the rule, he thought occasionally it did result, as in the present case—where there is no history of carcinoma in the family—from such applications.

Dr. W. F. Westmoreland was of the opinion that while cancer was generally hereditary, it was sometimes developed. He did not agree with Dr. Calhoun in this case, however; for it was local irritations, chronic, subacute in character, such as phymosis, and not acute inflammations that result from the application of escharotics or caustics, that give rise to this condition. The pterygium was more likely to have been the exciting cause; for the character of irritation it produces was an example of the local subacute inflammation that he alluded to, more than the applications.

Dr. Calhoun said that a large number of the people generally had a tendency to pterygium, which never developed into cancer.

Dr. J. G. Westmoreland: The seeds of cancer are present whenever a local trouble degenerates or assumes a cancerous condition.

Dr. W. F. Westmoreland: That proposition was still *sub-judice;* but there were in his opinion undoubtedly developed causes independent of diathesis. If the seeds were present in every case, operation would be useless in toto, and the cures that result not infrequently from surgical interference justify them.

Dr. Calhoun saw no more difficulty in believing that a cancerous diathesis could be acquired than that of tubercular, which is generally acknowledged. Some fungus growths, as for instance those frequently seen after an operation for strabismus, are aggravated, i.e., grow faster and larger by interference than when left alone.

Dr. Baird: The continual recurrence in Dr. Calhoun's case clearly pointed to a cancerous diathesis. Would like to know positively whether there ever was a case of cancer developed.

Dr. Jno. M. Johnson cited a case where a healthy man, without any history of carcinoma in his family, contracted a cancer from a cut on his upper lip while shaving. He had three plastic operations performed on him in Paris, the cancer returning after each one, and it finally killed him. He had been in the habit of shaving every day, and this was thought to be the source of irritation.

Dr. W. F. Westmoreland: That was an epithelial cancer.

Dr. Calhoun: Epithelial cancers are curable.

Dr. Westmoreland: When operated on before the glands become involved they are, but after these organs have become infiltrated with cancerous deposition from the epithelial variety, they are more malignant than any other variety; but if taken in time they will not return.

Dr. Johnson: So I am to understand that if they return the treatment was not begun soon enough?

Dr. Westmoreland: Yes; for an encephaloid or melanotic, if there be a diathesis, and the gland be not larger than a shot at the time of extirpation, will surely return; the epithelial not.

Dr. W. F. Westmoreland had under his care two cases of chorea. He desired the commiseration of the Academy, and wanted to know if any one knew of anything new in therapeutics of such cases. He had used arsenic with some advantage, but if there was a better remedy in the possession of any member he desired it.

Dr. Baird had had some experience with severe cases, and sketched an outline of his treatment, which had been highly satisfactory. When the case was very severe, the irregular contractions taking place with great violence, he would give chloroform by inhalation; keep the patient, as occasion required, under its influence for a greater or less length of time. Bromide of potassium in large doses, and chloral in conjunction, are useful. Tonics, both vegetable and mineral, and of the former he was much pleased with strychnia. He placed considerable stress upon and had much confidence in the application of ether spray to the spine, applied with an atomizer.

Dr. Jno. M. Johnson, while he approved of Dr. Baird's treatment, had used the spray to the spine, in one case, for six weeks, without benefit. He must have consumed five gallons of it in the various applications. She finally got well, and if anything other than time and nature brought about the cure, it was the valerinate of zinc and ferro-cyanate of quinine. She was under treatment for six months, and during that time he used every remedy he had ever read of, and all that he could hear suggested. As she got well on the two remedies mentioned above, he would use them in another case if opportunity presented. The cold douche to the head produced often a degree of composure which was much desired, and was to be recommended.

Dr. J. G. Westmoreland: From the physiological action of the calabar bean, he was inclined to believe, without having tried it, that it would be beneficial in cases of chorea. The cold shower bath had benefited a case of his very much.

Dr. W. F. Westmoreland asked Dr. Baird if he did not attribute much of the good effect of the ether spray to the spine to the inhalation of some portion of it.

Dr. Baird thought that its action was independent of any absorption.

Dr. Calhoun: A year and a half since, T. D. was pounding a copper cartridge, when it exploded, and a portion of the copper, very much the size and shape of the lachrymal bone, entered the front of the ball, causing a collapse of that organ, with complete blindness. The eye pained him severely, and enucleation was advised, to which however he would not consent. Since that time the eye has at intervals given much pain, rendering him unfit for work. A week since the injured eye was exquisitely painful, red, and very sensitive on pressure. The sound eye was inflamed, and vision in it failing; it was, in fact, sympathetically inflamed, and to save it the other was enucleated by Dr. J. W. Williams of this city, he performing the operation with dispatch and scientific ability, illustrating his thorough knowledge of the anatomy of the parts and his dexterity with the scalpel. The copper plate was found imbedded in the posterior portion of the globe. The customary dressings were applied, and the patient is doing finely, all sympathetic trouble having vanished.

Academy adjourned.

